# Supplementary material for: Association between pre-existing Pulmonary Hypertension and COVID-19 related outcomes in inpatient and ambulatory care settings
Source: PLoS One. 2025 Apr 24;20(4):e0321964. doi: 10.1371/journal.pone.0321964 (PMC12021216; doi:10.1371/journal.pone.0321964)
Supplement: S1 Data — (DOCX) [file pone.0321964.s001.docx]

**Supplemental Material:**

**Supplement 1:** Flow Chart for Selection of Inpatient vs. Outpatient Cohorts

**Supplement 2:** Test of Proportional-Hazards Assumption for Analysis of Outpatient Cohort

**Supplement 3:** Baseline Medications in the Inpatient Cohort

**Supplement 4:** Baseline Characteristics, Stratified by RVSP, in the Validation Cohort

**Supplement 5a:** Baseline Invasive Hemodynamics for Analysis of Inpatient Cohort

**Supplement 5b:** Baseline Invasive Hemodynamics for Analysis of Outpatient Cohort

**Supplement 6:** Baseline Echocardiographic Parameters of the Echocardiography Cohort

**Supplement 7:** Sample Size Stratified by PCWP and Relationship Between PH and In-Hospital Mortality Stratified by PCWP for Inpatient Cohort

**Supplement 8:** Relationship Between RVSP and Mortality Outcome in Validation Cohort

**Supplement 9:** Event rates for Outpatient Cohort, Split into Two Time Periods (0-90 Days, and 91-365 days)

**Supplement 10:** Outpatient Sample Size Stratified by PCWP and Relationship Between PH and Mortality for PCWP ≤ 15 mmHg in the Outpatient Cohort

**Supplement 1: Flow Chart for Selection of Inpatient vs. Outpatient Cohorts**

Veterans with a positive COVID test within four days of admission and/or up to 48 hours after admission

(n=1,204)

Negative test Only

(n=4,274)

Positive test only

(n=981)

Negative test and subsequent positive test

(n=1,321)

Veterans with at least one outpatient COVID test

(n=6,576)

Outpatient Cohort

Inpatient Cohort

Veterans with at least one COVID test (PCR or antigen) in the VA between 3/1/2020-8/31/2022

(n=17,484)

VHA enrolled Veterans 18+ years of age who underwent RHC with a recorded mean pulmonary artery pressure between 1/1/2016-12/31/2019

(n=41,353)

Veterans who did not have any COVID tests in the VA system

(n=23,869)

Veterans who only had a COVID test completed 3 days post admission and/or only had a negative COVID test in an inpatient setting.

(n=9,704)

**Supplement 2: Test of Proportional-Hazards Assumption for Analysis of Outpatient Cohort**


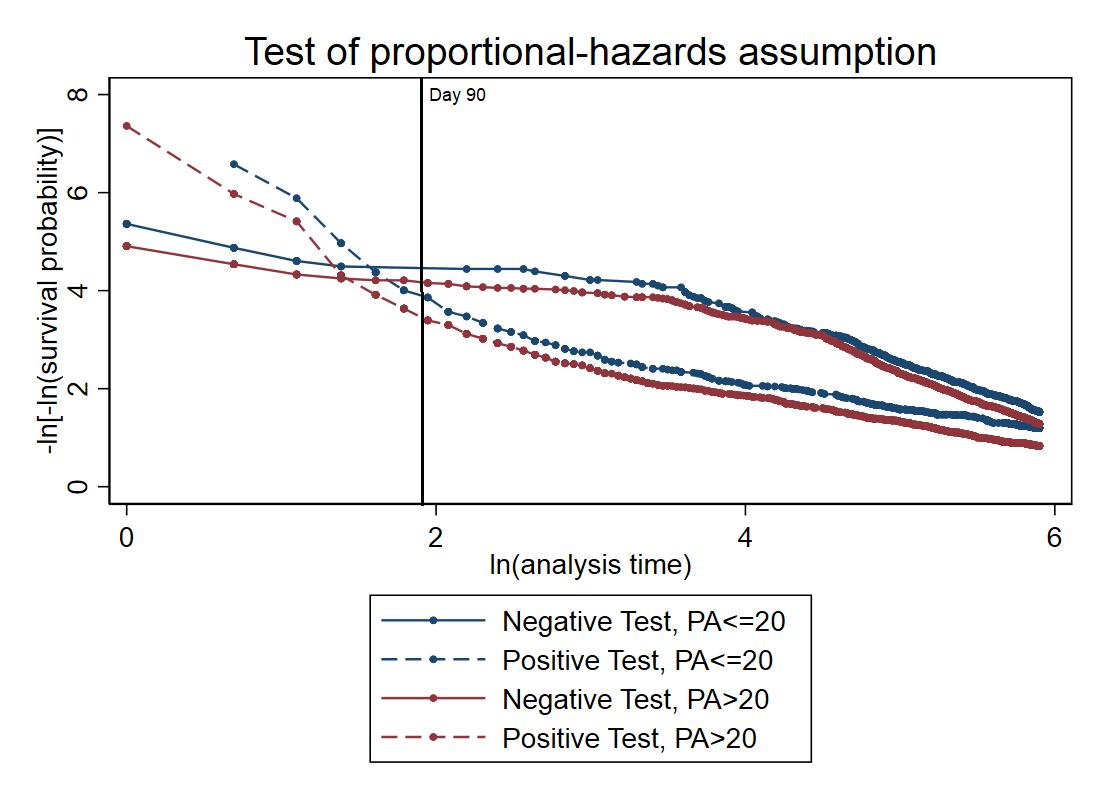


Graphical approach to testing proportional hazards assumption by plotting log-log Kaplan-Meier survival estimates against the log of analysis time. As seen in the above plot, the crossover time of the mortality curves was at 90 days.

**Supplement 3: Baseline Medications in the Inpatient Cohort**

|  | **Total**  **(n = 1204)** | **mPAP < 20 mmHg**  **(n = 252)** | **mPAP > 20 mmHg**  **(n = 952)** | **p-value** |
| --- | --- | --- | --- | --- |
|  |  |  |  |  |
| ACE Inhibitor | 511 (42.4%) | 110 (43.7%) | 401 (42.1%) | 0.66 |
| Anti-anginal | 434 (36.1%) | 91 (36.1%) | 343 (36.0%) | 0.98 |
| Anti-arrhythmic | 206 (17.1%) | 34 (13.5%) | 172 (18.1%) | 0.086 |
| Anticoagulant | 819 (68.0%) | 163 (64.7%) | 656 (68.9%) | 0.20 |
| Angiotensin II Receptor Blocker | 365 (30.3%) | 66 (26.2%) | 299 (31.4%) | 0.11 |
| Beta Agonist (Nebulizer) | 445 (37.0%) | 68 (27.0%) | 377 (39.6%) | <0.001 |
| Beta-blocker | 1008 (83.7%) | 201 (79.8%) | 807 (84.8%) | 0.056 |
| Bronchodilator | 634 (52.7%) | 117 (46.4%) | 517 (54.3%) | 0.026 |
| Calcium Channel Blocker | 469 (39.0%) | 95 (37.7%) | 374 (39.3%) | 0.65 |
| Furosemide | 777 (64.5%) | 127 (50.4%) | 650 (68.3%) | <0.001 |
| Hydrochlorothiazide | 91 (7.6%) | 24 (9.5%) | 57 (7.0%) | 0.18 |
| Inhaled Corticosteroids | 63 (5.2%) | 11 (4.4%) | 52 (5.5%) | 0.49 |
| Inhaled Budesonide | 16 (1.3%) | 5 (2.0%) | 11 (1.2%) | 0.31 |
| Inhaled Epoprostenol | 2 (0.2%) | 0 (0.0%) | 2 (0.2%) | 0.47 |
| Inhaled Nitric Oxide | 0 (0.0%) | 0 (0.0%) | 0 (0.0%) | - |
| Isoproterenol | 1 (0.1%) | 1 (0.4%) | 0 (0.0%) | 0.05 |
| Long-Acting Beta Agonist | 18 (1.5%) | 4 (1.6%) | 14 (1.5%) | 0.89 |
| Losartan | 254 (21.1%) | 41 (16.3%) | 213 (22.4%) | 0.035 |
| Valsartan | 146 (12.1%) | 29 (11.5%) | 117 (12.3%) | 0.74 |

Baseline medications taken prior to index hospitalization. Dichotomous variables presented as frequency (percentage) and analyzed using Chi-square testing.

Abbreviations: ACE = angiotensin-converting enzyme

**Supplement 4: Baseline Characteristics, Stratified by RVSP, in the Validation Cohort**

|  | **Total Cohort**  **(N=656)** | **RVSP < 30 (mmHg)**  **(n=261)** | **RVSP > 30 (mmHg)**  **(n=395)** | ***p*-value** |
| --- | --- | --- | --- | --- |
| Age (years)  Median (IQR) | 75 (65-84) | 72 (61-82) | 77 (68-86) | <0.001^1^ |
| BMI (kg/m^2^)  Median (IQR) | (n=535)  27.2 (23-32.8) | (n=213)  27.4 (23.6-32) | (n=322)  27 (22.7-32.9) | 0.55^1^ |
| Race  White  Black  Other | (n=654)  435 (66.5)  84 (12.8)  135 (20.6) | (n=260)  170 (65.4)  29 (11.2)  61 (23.5) | (n=394)  265 (67.3)  55 (13.9)  74 (18.8) | *Referent*  0.43^2^  0.21^2^ |
| Male Sex | 293 (44.7) | 129 (49.4) | 164 (41.5) | 0.046^2^ |
| Current smoker | (n=645)  54 (8.4) | (n=254)  19 (7.5) | (n=391)  35 (8.9) | 0.51^2^ |
| Hypertension | 539 (82.2) | 205 (78.5) | 334 (84.6) | 0.05^2^ |
| Hyperlipidemia | 400 (60.9) | 166 (63.6) | 234 (59.2) | 0.26^2^ |
| Diabetes mellitus | 288 (43.9) | 109 (41.8) | 179 (45.3) | 0.37^2^ |
| Atrial fibrillation/flutter | 236 (35.9) | 65 (24.9) | 171 (43.3) | <0.001^2^ |
| Chronic kidney disease | 181 (27.6) | 49 (18.8) | 132 (33.4) | <0.001^2^ |
| COPD | 91 (13.9) | 27 (10.3) | 64 (16.2) | 0.04^2^ |
| Obstructive sleep apnea | 62 (9.5) | 22 (8.4) | 40 (10.1) | 0.47^2^ |
| History of deep vein thrombosis | 38 (5.8) | 10 (3.8) | 28 (7.1) | 0.09^2^ |
| History of pulmonary embolism | 39 (5.9) | 11 (4.2) | 28 (7.1) | 0.13^2^ |
| Peripheral artery disease | 5 (0.76) | 0 (--) | 5 (1.3) | -- |
| Ischemic heart disease | 343 (52.3) | 113 (43.3) | 230 (58.2) | <0.001^2^ |
| Valvular heart disease | 82 (12.5) | 21 (8.1) | 61 (15.4) | 0.006^2^ |
| Congestive heart failure | 270 (41.2) | 68 (26.1) | 202 (51.1) | <0.001^2^ |
| Prior myocardial infarction | 117 (17.8) | 44 (16.9) | 73 (18.5) | 0.59^2^ |
| Prior TIA/stroke | 108 (16.5) | 42 (16.1) | 66 (16.7) | 0.84^2^ |
| Prior PCI | 68 (10.4) | 26 (9.9) | 42 (10.6) | 0.78^2^ |
| Prior CABG | 34 (5.2) | 7 (2.7) | 27 (6.8) | 0.02^2^ |

Abbreviations: BMI = body mass index; CABG = coronary artery bypass grafting; COPD = chronic obstructive pulmonary disease; HFpEF = heart failure with preserved ejection fraction; HFrEF = heart failure with reduced ejection fraction; PCI = percutaneous coronary intervention; TIA = transient ischemic attack.

*Categorical data are N (%)

+ includes ischemic heart disease, heart failure, myocardial infarction, prior PCI, and prior CABG

^1^Wilcoxon rank-sum

^2^Logistic regression; multinomial models used for variables with >2 categories

**Supplement 5a: Baseline Invasive Hemodynamics for Analysis of Inpatient Cohort**

|  | **Inpatient COVID-19 Positive, mPAP < 20 mmHg**  **(N=252)** | | **Inpatient COVID-19 Positive, mPAP > 20 mmHg**  **(N=952)** | |
| --- | --- | --- | --- | --- |
|  | **mean** | **% missing value** | **mean** | **% missing value** |
| RAP (mmHg) (SD) | 5.50  (3.03) | 0.00 | 11.30 (6.44) | 0.84 |
| PASP (mmHg) (SD) | 28.33  (5.73) | 0.00 | 48.18 (14.20) | 0.21 |
| PADP (mmHg) (SD) | 10.81  (4.05) | 0.00 | 21.73 (8.98) | 0.32 |
| PCWP (mmHg) (SD) | 10.26  (4.13) | 0.79 | 19.34 (7.45) | 3.78 |
| Cardiac Output Thermodilution (L/min)  (SD) | 5.62  (1.66) | 50.40 | 5.51 (1.91) | 47.48 |
| Cardiac Index by Thermodilution (L/min/m^2^)  (SD) | 2.68  (0.68) | 51.98 | 2.56 (0.85) | 48.42 |
| PAPi (SD) | 4.47  (3.96) | 2.78 | 3.22 (3.36) | 1.79 |
| RVSW (g/m/beat/m^2^) (SD) | 5.68  (2.11) | 86.11 | 10.11  (5.34) | 83.09 |

Abbreviations: mPAP = mean pulmonary artery pressure; PADP = pulmonary artery diastolic pressure; PAPi = pulmonary artery pulsatility index; PASP = pulmonary artery systolic pressure; PCWP = pulmonary capillary wedge pressure; RAP = right atrial pressure; RVSW = right ventricular stroke work

|  | **Negative Outpatient Covid Test, mPAP < 20 mmHg**  **(n=1766)** | | **Positive Outpatient Covid Test, mPAP < 20 mmHg**  **(n=721)** | | **Negative Outpatient Covid Test, mPAP > 20 mmHg**  **(n=3829)** | | **Positive Outpatient Covid Test, mPAP > 20 mmHg**  **(n=1581)** | |
| --- | --- | --- | --- | --- | --- | --- | --- | --- |
|  | **mean** | **% missing value** | **mean** | **% missing value** | **mean** | **% missing value** | **mean** | **% missing value** |
| RAP (mmHg) (SD) | 5.72  (26.86) | 0.23 | 5.00  (2.82) | 0.42 | 11.11 (19.11) | 0.55 | 11.71  (29.10) | 0.44 |
| PASP (mmHg) (SD) | 27.03  (5.02) | 0.28 | 26.89  (4.81) | 0.42 | 46.01 (13.76) | 0.29 | 45.92 (13.08) | 0.06 |
| PADP (mmHg) (SD) | 10.01  (4.27) | 0.51 | 10.08  (3.52) | 0.55 | 20.85 (7.21) | 0.29 | 20.92 (7.05) | 0.13 |
| PCWP (mmHg) (SD) | 9.42  (4.14) | 2.43 | 9.36  (3.61) | 1.53 | 18.86 (6.99) | 2.12 | 19.17 (7.30) | 2.66 |
| Cardiac Output Thermodilution (L/min)  (SD) | 5.39  (1.52) | 50.11 | 5.41  (1.45) | 48.82 | 5.73 (10.37) | 50.51 | 5.53  (1.72) | 51.55 |
| Cardiac Index by Thermodilution (L/min/m^2^)  (SD) | 2.61  (0.71) | 52.49 | 2.61  (0.67) | 50.49 | 2.58 (0.82) | 52.81 | 2.58  (0.81) | 53.64 |
| PAPi (SD) | 4.82  (4.51) | 3.45 | 2.80  (4.19) | 3.33 | 3.23 (5.89) | 1.04 | 3.07 (3.16) | 0.89 |
| RVSW (g/m/beat/m^2^) (SD) | 5.95  (2.65) | 80.12 | 6.09  (2.66) | 80.03 | 9.90  (4.81) | 79.08 | 8.56 (5.06) | 79.70 |

**Supplement 5b: Baseline Invasive Hemodynamics for Analysis of Outpatient Cohort**

Abbreviations: mPAP = mean pulmonary artery pressure; PADP = pulmonary artery diastolic pressure; PAPi = pulmonary artery pulsatility index; PASP = pulmonary artery systolic pressure; PCWP = pulmonary capillary wedge pressure; RAP = right atrial pressure; RVSW = right ventricular stroke work

**Supplement 6: Baseline Echocardiographic Parameters of the Validation Cohort**

|  | **Total Cohort**  **(N=656)** | **RVSP < 30 (mmHg)**  **(n=261)** | **RVSP > 30 (mmHg)**  **(n=395)** | **p-value** |
| --- | --- | --- | --- | --- |
| Echo time from index admission: |  |  |  |  |
| within 30 days | 29 (4.4) | 6 (2.3) | 23 (5.8) | 0.057 |
| 31-90 days prior | 75 (11.5) | 19 (7.3) | 56 (14.2) | 0.03 |
| 91-180 days prior | 62 (9.5) | 25 (9.6) | 37 (9.4) | 0.94 |
| 181-365 days prior | 94 (14.4) | 29 (11.1) | 65 (16.5) | 0.16 |
| 1 year prior | 151 (23.0) | 60 (22.9) | 91 (23.1) | *Referent* |
| 2 years prior | 94 (14.4) | 43 (16.5) | 51 (12.9) | 0.35 |
| ≥3 years prior | 150 (22.9) | 79 (30.3) | 71 (18.0) | 0.03 |
| **Echocardiography Parameter:** | | | | |
| RA Volume (ml)  (SD) | (n=290)  52.9 (31.9) | (n=94)  39.6 (21.1) | (n=196)  59.3 (34.2) | <0.001 |
| Estimated RA Pressure (mmHg)  (SD) | 5.4 (3.9) | 3.5 (1.8) | 6.6 (4.5) | <0.001 |
| TR Vmax (m/s)  (SD) | (n=613)  2.6 (0.48) | (n=235)  2.2 (0.28) | (n=378)  2.9 (0.39) | <0.001 |
| RVSP (mmHg)  (SD) | 34.5 (12.4) | 23.5 (4.5) | 41.7 (10.6) | <0.001 |
| IVC size (mm)  (SD) | (n=209)  18.4 (4.8) | (n=70)  16.5 (3.9) | (n=139)  19.3 (4.9) | <0.001 |
| IVC Inspiratory Collapse | (n=295) | (n=98) | (n=197) |  |
| Yes | 196 (66.4) | 84 (85.7) | 112 (56.9) | <0.001 |
| Dilated IVC | 20 (6.8) | 0 (--) | 20 (10.2) | 0.99 |
| N/A | 3 (1.0) | 1 (1.0) | 2 (1.0) | 0.48 |
| LVEF (%)  (SD) | (n=590)  58.5 (11.6) | (n=227)  59.5 (9.7) | (n=363)  57.9 (12.6) | 0.91 |
| Estimated Pulmonary Artery Systolic Pressure (mmHg)  (SD) | (n=466) | (n=157) | (n=309) |  |
| Normal | 262 (56.2) | 149 (94.9) | 113 (36.6) | 0.78 |
| Unable to Estimate | 4 (0.86) | 2 (1.3) | 2 (0.65) | 0.98 |
| Mild | 123 (26.4) | 0 (--) | 123 (39.8) | 0.99 |
| Moderate | 46 (9.9) | 0 (--) | 46 (14.9) | *Referent* |
| Severe | 26 (5.6) | 6 (3.8) | 20 (6.5) | 0.002 |
| TAPSE (mm)  (SD) | (n=529)  20.3 (4.6) | (n=215)  20.5 (4.3) | (n=314)  20.2 (4.8) | 0.38 |
| MV E/e’ | (n=300)  13.0 (6.0) | (n=122)  11.3 (4.3) | (n=178)  14.3 (6.7) | <0.001 |
| LAVI (ml/m^2^)  (SD) | (n=531)  35.8 (15.8) | (n=200)  30.4 (12.7) | (n=331)  39.1 (16.6) | <0.001 |

Continuous variables presented as mean (standard deviation) and analyzed using T-test, and dichotomous variables presented as frequency (percentage) and analyzed using Chi-square testing.

Abbreviations: IVC = inferior vena cava; LAVI = left atrial volume index; LVEF = left ventricular ejection fraction; MV E/e’ = mitral valve early inflow velocity (E) over the early diastolic mitral annular velocity (e’); RA = right atrial; RVSP = right ventricular systolic pressure; TAPSE = tricuspid annular plane systolic excursion; TR = tricuspid regurgitation; TVPG = tricuspid valve pressure gradient

**Supplement 7: Inpatient Sample Size Stratified by PCWP and Relationship Between PH and In-Hospital Mortality Stratified by PCWP**

|  | **PCWP ≤ 15 mmHg** | | **PCWP >15 mmHg** | |
| --- | --- | --- | --- | --- |
|  | N | % | N | % |
| No PH (mPAP < 20 mmHg) | 212 | 45.79 | 40 | 5.40 |
| PH (mPAP > 20 mmHg) | 251 | 54.21 | 701 | 94.60 |
|  |  |  |  |  |
|  |  | **OR** | **97.5% CI** | **p-value** |
| **Outcome: In Hospital Mortality** |  |  |  |  |
| mPAP > 20 mmHg vs. < 20 mmHg | PCWP ≤ 15 | 1.93 | (1.07, 3.46) | 0.029 |
|  | PCWP >15 | 1.24 | (0.48, 3.20) | 0.654 |

Abbreviations: mPAP = mean pulmonary artery pressure; PCWP: pulmonary capillary wedge pressure; PH = pulmonary hypertension

Logistic regression adjusted for demographic age, sex, race, marital status, history of myocardial infarction, congestive heart failure, peripheral vascular disease, cerebrovascular accident or transient ischemic attack, dementia, chronic obstructive pulmonary disease, connective tissue disease, peptic ulcer disease, liver disease, diabetes mellitus, moderate to severe chronic kidney disease, solid tumor, leukemia, lymphoma, acquired immunodeficiency syndrome), and number of hospitalizations and VA emergency department visits in the one year preceding the positive COVID-19 test with time and region fixed effects, clustered at the patient level.

**Supplement 8. Relationship Between RVSP and Mortality Outcome in Validation Cohort**

|  | **Exposure** | **Odds Ratio** | **95% CI** | ***p*-value** |
| --- | --- | --- | --- | --- |
| Mortality during hospital stay | RVSP > 30 mmHg vs. < 30 mmHg | 2.12 | (1.18, 3.82) | 0.01 |
|  | RVSP Continuous | 1.10 | (0.90, 1.22) | 0.63 |

Abbreviations: cPAP = continuous positive airway pressure; ICU = intensive care unit; MI = myocardial infarction

Logistic regression adjusted for age, current tobacco use, sex, chronic kidney disease, cerebrovascular disease, chronic obstructive pulmonary disease, cardiovascular events, congestive heart failure, diabetes mellitus, hypertension, race, body mass index, and time from index echocardiogram to hospitalization.

RVSP continuous modeled as per 10 mmHg increase in RVSP.

**Supplement 9: Summary of Event rates for Outpatient Cohort, Split into Two Time Periods (0-90 Days, and 91-365 days)**

|  | **Negative Outpatient COVID-19 Test, mPAP ≤ 20 mmHg**  **(n=1766)** | **Positive Outpatient COVID-19 Test, mPAP ≤ 20 mmHg**  **(n=721)** | **Negative Outpatient COVID-19 Test, mPAP > 20 mmHg**  **(n=3829)** | **Positive Outpatient COVID-19 Test, mPAP > 20 mmHg**  **(n=1581)** | **p-value** |
| --- | --- | --- | --- | --- | --- |
| Died within 90 days | 14 (0.79%) | 34 (4.72%) | 37 (0.97%) | 99 (6.26%) | <0.001 |
| Hospitalized within 90 days | 114 (6.46%) | 71 (9.85%) | 284 (7.42%) | 213 (13.47%) | <0.001 |
| Death or Hospitalization within 90 days | 128 (7.25%) | 99 (13.73%) | 320 (8.36%) | 291 (18.41%) | <0.001 |
|  |  |  |  |  |  |
| *Only including those who survived to day 91* | **Negative Outpatient COVID-19 Test, mPAP ≤ 20**  **mmHg**  **(n=1752)** | **Positive Outpatient COVID-19 Test, mPAP ≤ 20**  **mmHg**  **(n=687)** | **Negative Outpatient COVID-19 Test, mPAP > 20**  **mmHg**  **(n=3792)** | **Positive Outpatient COVID-19 Test, mPAP > 20**  **mmHg**  **(n=1482)** | **p-value** |
| Died between 91-365 days | 31 (1.77%) | 17 (2.47%) | 132 (3.48%) | 43 (2.90%) | <0.001 |
| Hospitalized between 91-365 days | 220 (12.56%) | 64 (9.32%) | 572 (15.08%) | 225 (15.18%) | <0.001 |
| Death or Hospitalization between 91-365 days | 240 (13.70%) | 77 (11.21%) | 665 (17.54%) | 256 (17.27%) | <0.001 |

Dichotomous variables presented as frequency (percentage) and analyzed using Chi-square testing.

Abbreviations: mPAP = mean pulmonary artery pressure

**Supplement 10:** **Outpatient Sample Size Stratified by PCWP and Relationship Between PH and Mortality for PCWP ≤ 15 mmHg in the Outpatient Cohort**

|  | **PCWP ≤ 15 mmHg** | | **PCWP > 15 mmHg** | |
| --- | --- | --- | --- | --- |
|  | **N** | **%** | **N** | **%** |
| Negative Outpatient COVID-19 Test, mPAP ≤ 20 mmHg | 1639 | 39.44 | 127 | 3.39 |
| Positive Outpatient COVID-19 Test, mPAP ≤ 20 mmHg | 681 | 16.39 | 40 | 1.07 |
| Negative Outpatient COVID-19 Test, mPAP 21+ mmHg | 1313 | 31.59 | 2516 | 67.25 |
| Positive Outpatient COVID-19 Test, mPAP 21+ mmHg | 523 | 12.58 | 1058 | 28.28 |

|  |  | **0-90 days** | | | **91-365 days** | | |
| --- | --- | --- | --- | --- | --- | --- | --- |
|  |  | **HR** | **97.5% CI** | **p-value** | **HR** | **97.5% CI** | **p-value** |
| **Outcome: Mortality** | **PCWP** |  |  |  |  |  |  |
| COVID-19 Positive, No PH vs. COVID-19 Negative, No PH | **≤**15 mmHg | 8.98 | (4.04, 19.89) | <0.001 | 3.28 | (1.64, 6.59) | <0.001 |
| COVID-19 Negative, PH vs. COVID-19 Negative, No PH | **≤**15 mmHg | 1.35 | (0.57, 3.16) | 0.432 | 1.72 | (1.03, 2.88) | 0.018 |
| COVID-19 Positive, PH vs. COVID-19 Negative, No PH | **≤**15 mmHg | 10.30 | (4.53, 23.41) | <0.001 | 3.29 | (1.67, 6.50) | <0.001 |

Abbreviations: mPAP = mean pulmonary artery pressure; PCWP = pulmonary capillary wedge pressure; PH = pulmonary hypertension

Cox-regression adjusted for demographic age, sex, race, marital status and history of myocardial infarction, congestive heart failure, peripheral vascular disease, cerebrovascular accident or transient ischemic attack, dementia, chronic obstructive pulmonary disease, connective tissue disease, peptic ulcer disease, liver disease, diabetes mellitus, moderate to severe chronic kidney disease, solid tumor, leukemia, lymphoma, acquired immunodeficiency syndrome, and number of hospitalizations and VA emergency department visits in the one year preceding the positive COVID-19 test with time and region fixed effects, clustered at the patient level.
